# Melatonin in crop plants: from biosynthesis through pleiotropic effects to enhanced stress resilience

**DOI:** 10.1007/s13353-025-00963-7

**Published:** 2025-04-30

**Authors:** Martyna Michałek, Piotr Ogrodowicz, Michał Kempa, Anetta Kuczyńska, Krzysztof Mikołajczak

**Affiliations:** Institute of Plant Genetics, Polish Academy of Sciences, Strzeszyńska 34, 60-479 Poznań, Poland

**Keywords:** Antioxidant systems, Crop resilience, Melatonin, Melatonin derivatives, Phytohormones, Root growth

## Abstract

Melatonin plays a crucial role in enhancing plant resilience to environmental stresses by regulating physiological and biochemical responses. This review provides an overview of melatonin biosynthesis, signaling pathways, and its interactions with phytohormones, highlighting its multifunctional roles across various crop species. We summarize recent discoveries regarding the biosynthetic pathways of melatonin and its crucial metabolites, emphasizing the importance of tryptophan and serotonin in this process. Furthermore, we discuss the intricate crosstalk between melatonin and phytohormones, particularly auxins, cytokinins, and brassinosteroids, which collectively influence root development, growth, and stress tolerance, among other traits. The antioxidant activity of melatonin and its derivatives, along with their impact on photosynthesis, has also been thoroughly discussed. Notably, melatonin’s regulatory actions promote root growth, thereby improving water and nutrient absorption under stress conditions. The identification of candidate genes and a putative receptor provides a foundation for future studies aimed at elucidating the molecular mechanisms underlying melatonin signaling in crop species. Ultimately, this review underscores the potential of harnessing melatonin in crop improvement strategies to enhance resilience to abiotic stresses while promoting sustainable agricultural practices.

## Introduction

To mitigate the effects of stress, plants have developed a variety of adaptive mechanisms, including the production of biomolecules that modulate physiological and biochemical responses (Khan et al. 2022). Among these regulatory molecules, melatonin (N-acetyl-5-methoxytryptamine) has garnered significant attention in recent years (Zeng et al. 2022). According to the Scopus database (1995–2024), over half of the reports on melatonin in agricultural and biological sciences have been published in the past 5 years (Scopus.com, accessed June 2024). Melatonin’s multidirectional action in plants, including its regulation of gene expression, enhancement of antioxidant defense systems, and interactions with phytohormones, allows plants to fine-tune metabolic processes and effectively respond to stress signals (Arnao and Hernández-Ruiz 2021). Notably, melatonin’s regulation of phytohormone networks, particularly brassinosteroid (BR) signaling pathways, holds vast potential for increasing plant tolerance to environmental stresses. BRs play a crucial role in regulating root development, a key factor for survival in water-limited environments (Sun et al. 2021; Wei and Li 2016).

This review summarizes recent advances in understanding melatonin biosynthesis, metabolism, signaling, and functions across various crop species. Moreover, it discusses the impact of melatonin on plant growth, development, and stress tolerance, with a special focus on its role in hormonal regulation. Additionally, it explores the dynamic interaction between melatonin and phytohormones in regulating root growth, offering valuable insights into potential strategies for crop improvement.

## Melatonin biosynthesis and catabolism

**Fig. 1:**
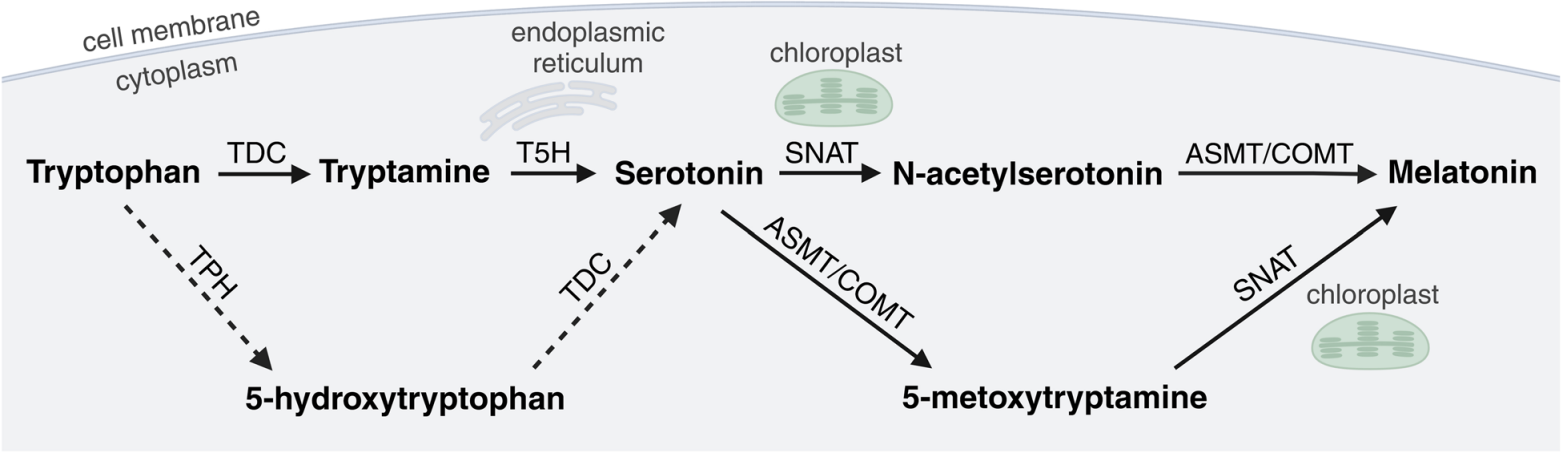
Melatonin biosynthesis in plants. Dashed arrows indicate hypothetical biosynthetic pathways. TDC, tryptophan decarboxylase; TPH, tryptophan hydroxylase; T5H, tryptamine 5-hydroxylase; SNAT, serotonin N-acetyltransferase; ASMT, N-acetylserotonin methyltransferase; COMT, caffeic acid O-methyltransferase (figure drafted on the base of Yu et al. 2018; Khan et al. 2023a, using BioRender tool at http://biorender.com)

Melatonin biosynthesis begins with tryptophan, which is initially converted to serotonin through a two-step process involving decarboxylation and hydroxylation (Back et al. 2016; Erland 2024). In the cytoplasm, tryptophan decarboxylase (TDC) converts tryptophan into tryptamine, which is then hydroxylated to serotonin by tryptamine 5-hydroxylase (T5H), an enzyme located in the endoplasmic reticulum (Fig. 1). Subsequently, serotonin is converted into N-acetylserotonin by serotonin N-acetyltransferase (SNAT), an enzyme primarily found in chloroplasts. Melatonin is then synthesized from N-acetylserotonin by cytosolic N-acetylserotonin methyltransferase (ASMT) or caffeic acid O-methyltransferase (COMT) (Xie et al. 2022). COMT, an enzyme with a broad substrate spectrum, exhibits low specificity for N-acetylserotonin (Wang et al. 2019). Therefore, COMT-mediated methylation of N-acetylserotonin is limited by the enzyme’s preference for other substrates, allowing ASMT to play a dominant role in this process (Byeon et al. 2015a; Back et al. 2016; Huang et al. 2024). Nonetheless, numerous studies on crop plants, including *Oryza sativa* (Huangfu et al. 2022), *Citrullus lanatus* (Chang et al. 2021), *Nicotiana tabacum* (Yao et al. 2022), *Gossypium hirsutum* L. (Zhang et al. 2024), *Solanum lycopersicum* L. (Ahammed et al. 2019; Sun et al. 2020a), and *Camellia sinensis* (Pham et al. 2024), highlight the significance of COMT in melatonin synthesis.

Under stress conditions or during plant senescence, when serotonin accumulates to high levels, it can be converted to 5-methoxytryptamine by ASMT/COMT and subsequently to melatonin by SNAT, causing the final step of biosynthesis to occur in chloroplasts (Fig. 1) (Choi et al. 2017; Lee et al. 2014; Liu et al. 2022a). Melatonin production may also take place in other cellular compartments. For example, mitochondria isolated from *Arabidopsis thaliana* with a *SNAT* isoform cloned from *Malus zumi* retained the ability to produce melatonin after serotonin treatment (Wang et al. 2017). However, other studies indicate that SNATs are mainly localized in chloroplasts (Byeon et al. 2014; Yu et al. 2019). Tan and Reiter (2020) propose that under stress conditions, melatonin synthesis could shift from chloroplasts to mitochondria when the former is compromised.

Melatonin synthesis pathway in plants shares certain elements with those found in other organisms (Zhao et al. 2019a). In both plants and animals, melatonin synthesis begins with tryptophan, which is converted to serotonin; however, the intermediates and enzymes involved in these processes differ significantly. In animals, the pathway initiates with the conversion of tryptophan into 5-hydroxytryptophan by tryptophan hydroxylase (TPH), followed by transformation into serotonin via aromatic L-amino acid decarboxylase. Subsequently, serotonin is converted into N-acetylserotonin by SNAT or arylamine N-acetyltransferase (AANAT), and finally, N-acetylserotonin is converted into melatonin by ASMT or hydroxyindole-O-methyltransferase (Kim et al. 2024). The biosynthetic pathway of melatonin in bacteria is expected to be analogous to that in animals, following the 5-hydroxytryptophan route. For instance, Ma et al. (2017) revealed that in *Pseudomonas fluorescens*, isotopic tryptophan was incorporated into 5-hydroxytryptophan, but not into tryptamine. Similarly, Jiao et al. (2016) reported that *Bacillus amyloliquefaciens* produced various melatonin intermediates, including 5-hydroxytryptophan, but not tryptamine. In contrast, Muñiz‐Calvo et al. (2019) found that in *Saccharomyces cerevisiae*, tryptophan is converted directly into tryptamine without undergoing hydroxylation. Additionally, *S. cerevisiae* possesses enzymes such as AANAT and polyamine acetyltransferase, which, along with the detection of 5-methoxytryptamine as an intermediate, suggest that in yeasts melatonin may be synthesized directly from this compound (Bisquert et al. 2023; Que et al. 2020).

Notably, there may be an alternative route for melatonin synthesis in plants that bypasses the involvement of T5H. In rice mutants lacking T5H activity, which blocks serotonin production, both melatonin and 5-hydroxytryptophan, an intermediate in serotonin synthesis in animals, were found to increase (Park et al. 2012). These findings suggest the possible existence of a similar pathway in plants, although TPH, the enzyme responsible for 5-hydroxytryptophan production, has not yet been identified in plants (Tan et al. 2016a). For *TPH1* and *TPH2* genes, which are highly conserved among vertebrates (Xu et al. 2019), we applied a sequence alignment-based method (www.blast.ncbi.nlm.nih.gov); however, no homologs were detected in monocots such as *Triticum aestivum* L. and *Hordeum vulgare* L. (Table 1), nor in the dicot *Glycine max* L. These species were selected for analysis as representatives of economically important crops with complex genomes. It has been proposed that a putative TPH-like in plants could generate 5-hydroxytryptophan, which is then converted into serotonin by TDC (Park et al. 2008; Mannino et al. 2021). While direct evidence of TPH as a functional enzyme in plants is lacking, the existence of alternative pathways for melatonin biosynthesis suggests a complexity that warrants further investigation. Moreover, there may be an as-yet-undiscovered melatonin biosynthesis pathway that bypasses serotonin entirely. Arnao et al. (2023) proposed a melatonin biosynthesis route involving SNAT, which converts tryptamine to N-acetyltryptamine. Subsequently, N-acetyltryptamine can be converted into N-acetylserotonin by T5H, although this pathway is yet to be confirmed in plants. To date, SNAT-mediated production of N-acetyltryptamine was experimentally validated in the cyanobacterium *Synechocystis* sp. by Byeon et al. (2013). The potential SNAT-mediated conversion of tryptamine in plants requires further research, as it could eliminate the inhibitory effect of serotonin on melatonin production (Back et al. 2016).

**Table 1:** Genes encoding enzymes involved in melatonin biosynthesis and degradation in *O. sativa* and their potential orthologs in cereal species

	**No**	**Enzyme**	***Oryza sativa* gene**^**a**^	***Hordeum vulgare* ortholog ID (Genbank)**	***Triticum aestivum* ortholog ID (Genbank)**
Biosynthetic pathway	1	Tryptophan decarboxylase (TDC)	*OsTDC1* (AK069031)	AB162961; AB162960; XM_045114866; XM_045114864; XM_045099112; XM_045114865; XM_045115971	MH891155; GU817319; XM_044508239; XM_044508227; XM_044581723; XM_044505149; XM_044473192; XM_044571762; XM_044587040; XM_044571760; XM_044586654; XM_044570001; XM_044496723; XM_044511356
			*OsTDC2* (AK103253)	AK361266; AK360932; XM_045090812; XM_045090813; XM_045090814	BT009489; XM_044524509; XM_044524510; XM_044531923; XM_044531924; XM_044531925; XM_044540238; XM_044540239; XM_044540240; XM_044540241; XR_006449595
			*OsTDC3* (AK065830)	AK367736; AK248813; AK363410; XM_045116881; XM_045098135; XM_045098129	AK454808; XM_044481329; XM_044488957; XM_044502047; XM_044580830; XM_044496877; XM_044522625; XM_044590676; XM_044590595
	2	Tryptamine 5-hydroxylase (T5H)	*OsT5H* (AK071599)	AK368713; XM_045096456	XM_044560344; XM_044553149; XM_044550226; XM_044552661; XM_044511412; XM_044522692
	3	Serotonin N-acetyltransferase (SNAT)	*OsSNAT1* (AK059369)	AK249545; XM_045121374; XR_006631012; XR_006631010	XM_044556727; XM_044482787; XM_044594922; XR_006467767
			*OsSNAT2* (AK068156)	AK360456; AK359698; AK354655; AK353661; XM_045101717	XM_044586903; XM_044566956
	4	N-acetylserotonin methyltransferase (ASMT)	*OsASMT1* (AK072740)	XM_045109144	XM_044471694; XM_044505515; XM_044554282; XM_044463359; XM_044591754; XM_044505914; XM_044505514; XM_044595926
			*OsASMT2* (AK069308)	XM_045109144	XM_044463359; XM_044471694; XM_044505914; XM_044505514; XM_044591754; XM_044505515; XM_044554282
			*OsMTS1*^*b*^ (LOC_Os07g14350)	AK356252; AK250254; AK374149; XM_045090578; XM_045090579	XM_044540109; XM_044531788; XM_044524426; XM_044540110; XM_044531789; XM_044531790; XM_044540111
	5	Caffeic acid O-methyl transferase (COMT)	*OsCOMT* (AK064768)	AK359402; AK248737; AK373043; AK251181; EF586876; AK248293; AK364734; AK367297; AK373701; AK363914; XM_045103585; XM_045122769; XM_045096120; XM_045115764; XM_045115029; XM_045102846	AY226581; BT009383; EF413031; DQ223971; AK448629; AK332908; MT157512; MT166264; EF423610; EF423611; ON108664; XM_044567041; NM_001427990; XM_044582561; NM_001405843; XM_044487643; XM_044504009; XM_044554430; XM_044559953; XM_044549872; XM_044585329; XM_044473989; XM_044589266; XM_044585932; XM_044543988; XM_044473987; XM_044465746
	6	Tryptophan hydroxylase (TPH)	none	none	none
Degradation	6	Melatonin 2-hydroxylase (M2H)	*Os2-ODD11*^*c*^ (AK067086)	XM_045116353; AK368601; AK249480	XM_044516010; AK455157; XM_044475829; AK336120
			*Os2-ODD19* (AK065790)	AK355337; AK358416; XM_045090469; XM_045121356; XM_045121355	AK335236; XM_044539213; XM_044539214; XM_044523521
			*Os2-ODD21* (AK119413)	XM_045114998	XM_044473291; AK448699; XM_044598235; XM_044465027; XM_044479823
			*Os2-ODD33*^*c*^ (AK101447)	AK248549; AK356484; AK249298; XM_045100544; XM_045100545	AK456027; XM_044577521; XM_044568370; XM_044585490; XM_044568371
	7	Melatonin 3-hydroxylase (M3H)	*Os2-ODD11* (AK067086)	XM_045116353; AK368601; AK249480	XM_044516010; XR_006463441; AK455157; XM_044475829; AK336120
			*Os2-ODD26* (AK071626)	AK373625; XM_045117196	XM_044495393; XM_044481617; XM_044503032; XM_044481618; XM_044504058
			*Os2-ODD33* (AK101447)	AK248549; AK356484; AK249298; XM_045100544; XM_045100545	AK456027; XM_044577521; XM_044585490; XM_044568370; XM_044568371
	8	Indoleamine-2,3-dioxygenase (IDO)	*OsIDO* (AK110021)	None	None

In the National Center for Biotechnology Information database, accession numbers beginning with the ‘XM’ prefix represent mRNA transcripts predicted by gene prediction algorithms during the genome annotation process (http://www.ncbi.nlm.nih.gov). A complete list of gene accession prefixes can be found in the DNA Data Bank of Japan database at http://www.ddbj.nig.ac.jp/documents/prefix-e

^a^Genes involved in melatonin biosynthesis and catabolism reported by Okazaki et al. (2010), Byeon et al. (2015a, 2016), Lee et al. (2016), Wei et al. (2016), Hong et al. (2018)

^b^O-methyltransferase with ASMT activity, according to Hong et al. (2018)

^c^Os2-ODD11 and Os2-ODD33 with proven M2H and M3H activity by Lee et al. (2016), Wei et al. (2016)

### Enzymatic and non-enzymatic conversion of melatonin in plants

**Fig. 2:**
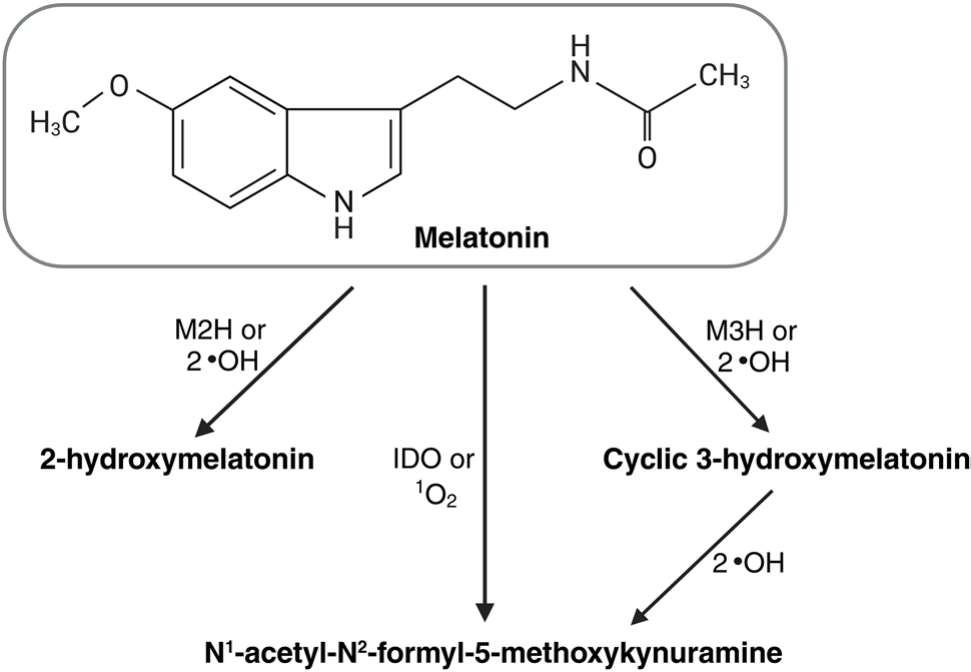
Main melatonin metabolites in plants. M2H, melatonin 2-hydroxylase; M3H, melatonin 3-hydroxylase; IDO, indoleamine-2,3-dioxygenase; ^1^O_2_, singlet oxygen; ·OH, hydroxyl radical (figure drafted on the base of Hardeland 2017; Back 2021, using BioRender tool at http://biorender.com)

The melatonin molecule possesses methoxy and acetyl side chains, which contribute to its amphiphilic nature, allowing it to easily cross biological membranes and access various compartments within plant cells. Once in specific cellular compartments, melatonin can undergo both enzymatic and non-enzymatic transformations (Mannino et al. 2021). The primary products of melatonin’s enzymatic modifications in plants include its hydroxylated derivatives: 2-hydroxymelatonin (2-OHM) and cyclic 3-hydroxymelatonin (C3-OHM), followed by the metabolite N^1^-acetyl-N^2^-formyl-5-methoxykynuramine (AFMK) (Fig. 2).

The enzymes responsible for melatonin conversion are distributed across different cellular compartments. Melatonin 2-hydroxylase (M2H), which produces 2-OHM, is mainly located in chloroplasts, while melatonin 3-hydroxylase (M3H) and indoleamine-2,3-dioxygenase (IDO), which generate C3-OHM and AFMK, respectively, are observed in the cytoplasm. Both M2H and M3H exhibit higher catalytic efficiency in plants compared to melatonin biosynthetic enzymes such as SNAT and ASMT (Hardeland 2017; Back 2021). A quantitative analysis of 2-OHM in 24 plant species, including 11 crop plants, revealed that its average content was significantly higher than that of melatonin (Byeon et al. 2015b). Lee et al. (2016) further demonstrated that the catalytic efficiency of M3H in *O. sativa* exceeded that of M2H, resulting in C3-OHM levels more than tenfold higher than 2-OHM. In contrast, AFMK was produced at a considerably lower level compared to the two previously mentioned melatonin derivatives. These findings indicate that melatonin undergoes rapid metabolism within plant cells, predominantly forming 2-OHM and C3-OHM.

The same metabolites can also form through the non-enzymatic transformation of melatonin caused by free radicals and other reactive compounds generated during oxidative stress. Melatonin has a high reduction potential due to its electron-rich indole ring, enabling it to react with various oxygen- and nitrogen-based free radicals in plant cells. For instance, hydroxylation of melatonin via reactions with two hydroxyl radicals can occur at any unsubstituted carbon atom on the indole ring, leading to the formation of metabolites such as 2-OHM and C3-OHM. AFMK can then be produced when C3-OHM reacts with two hydroxyl radicals (Hardeland 2016). However, studies by Lee et al. (2016) revealed that treating *O. sativa* with C3-OHM did not result in AFMK production, suggesting that in plants, AFMK may be primarily synthesized from melatonin via IDO or alternative pathways. Hardeland (2017) indicated that AFMK can be formed through various non-enzymatic processes, either from melatonin itself or through its metabolites. One such pathway involves the direct reaction of melatonin with singlet oxygen (Fig. 2). Furthermore, melatonin-derived metabolites are likely formed under ultraviolet radiation (UVR) exposure, as the absorption of UVR by melatonin’s aromatic indole ring may induce chemical bonds cleavage and structural transformations. In a cell-free system, it was demonstrated that UVR induces melatonin catabolism, leading to the formation of derivatives such as AFMK and 2-OHM (Fischer et al. 2006). UVR also excites various cellular components, transferring energy to molecular oxygen and generating singlet oxygen, which then reacts with melatonin. As UVR influences the melatonin molecule both directly and indirectly, its metabolism is promoted during light exposure (Pospíšil et al. 2019; Slominski et al. 2017a, 2018). Notably, tryptophan, similar to melatonin, can also undergo conversion by UVR and reactive oxygen species (ROS), with 5-hydroxytryptophan as a product (Slominski et al. 2012; Bellmaine et al. 2020). The nonenzymatic synthesis pathway of 5-hydroxytryptophan could explain the existence of an alternative melatonin synthesis pathway via 5-hydroxytryptophan, despite the absence of the TPH enzyme in plants.

### Pleiotropic effects of melatonin derivatives

Melatonin transformation to its derivatives leads to biologically relevant effects. For instance, the antioxidant properties of AFMK  have been widely described in human studies. It has been shown that AFMK itself is not as potent antioxidant as melatonin; however, its derivative, N^1^-acetyl-5-methoxykynuramine, exhibits stronger antioxidative effects (Hardeland 2017; Janjetovic et al. 2014; Tan et al. 2015; Bocheva et al. 2022). However, it remains unclear whether AFMK plays a significant role in plant antioxidant defense. Tan et al. (2007) observed that higher levels of AFMK were formed in *Eichhornia crassipes* plants exposed to high light intensity, suggesting its potential role in photoprotection against UVR. In contrast, metabolites such as 2-OHM and C3-OHM have been studied in more detail. Lee and Back (2022) demonstrated that C3-OHM exhibits significantly higher antioxidant activity than melatonin and 2-OHM in *A. thaliana*. This finding aligns with the earlier prediction by Pérez-González et al. (2017) regarding the low antioxidant activity of 2-OHM, based on its structural features. According to Korkmaz et al. (2023), 2-OHM reduces oxidative damage in *Capsicum annuum* L. by increasing proline levels and the activity of antioxidant enzymes. A similar effect was observed in *Cucumis sativus* L., where 2-OHM induced antioxidants accumulation and the expression of stress-responsive genes (Shah et al. 2019). C3-OHM, beyond its antioxidant properties, has been linked to the regulation of flowering-related gene expression in *A. thaliana* (Lee and Back 2022). These findings indicate that the pleiotropic effects of melatonin in plants are likely not only due to the properties of melatonin itself, but also result from its derivatives.

### In silico identification of melatonin biosynthetic and catabolic genes in crop plants

Knowledge about melatonin synthesis and degradation in plants primarily derives from studies on *O. sativa* (Back 2021). Orthologs of *O. sativa* melatonin biosynthetic genes have been identified in silico for various plant species (Bhowal et al. 2021; Yang et al. 2022); however, catabolic genes remain largely unexplored in many crops, including cereals. In this study, using experimentally confirmed *O. sativa* gene sequences involved in melatonin biosynthesis and catabolism, along with a sequence alignment-based method (http://www.blast.ncbi.nlm.nih.gov), we identified their putative orthologs in *H. vulgare* and *T. aestivum* (Table 1). Sequences were filtered based on the criteria of over 50% query coverage and 70% identity. For melatonin catabolic genes encoding 2-oxoglutarate-dependent dioxygenases, 2-ODD11, which exhibits both M2H and M3H activities, and 2-ODD21, with M3H activity (Lee et al. 2016; Wei et al. 2016), query coverage was lowered to 23% to facilitate the identification of potential orthologs.

No orthologs of the rice gene encoding the IDO enzyme were found in either *H. vulgare* or *T. aestivum* (Table 1). The presence of signature domains (pyridoxal-dependent decarboxylase, PF00282; cytochrome P450, PF00067; acetyltransferase, PF00583; O-methyltransferase domains, PF08100 and PF00891; leucine carboxyl methyltransferase, PF04072; 2-oxoglutarate-dependent dioxygenase domains, PF14226 and PF03171) confirmed the putative identity of the identified orthologs. All candidate genes identified through in silico analysis require further research to confirm their functional roles in melatonin biosynthesis and degradation.

## Melatonin signaling

**Fig. 3:**
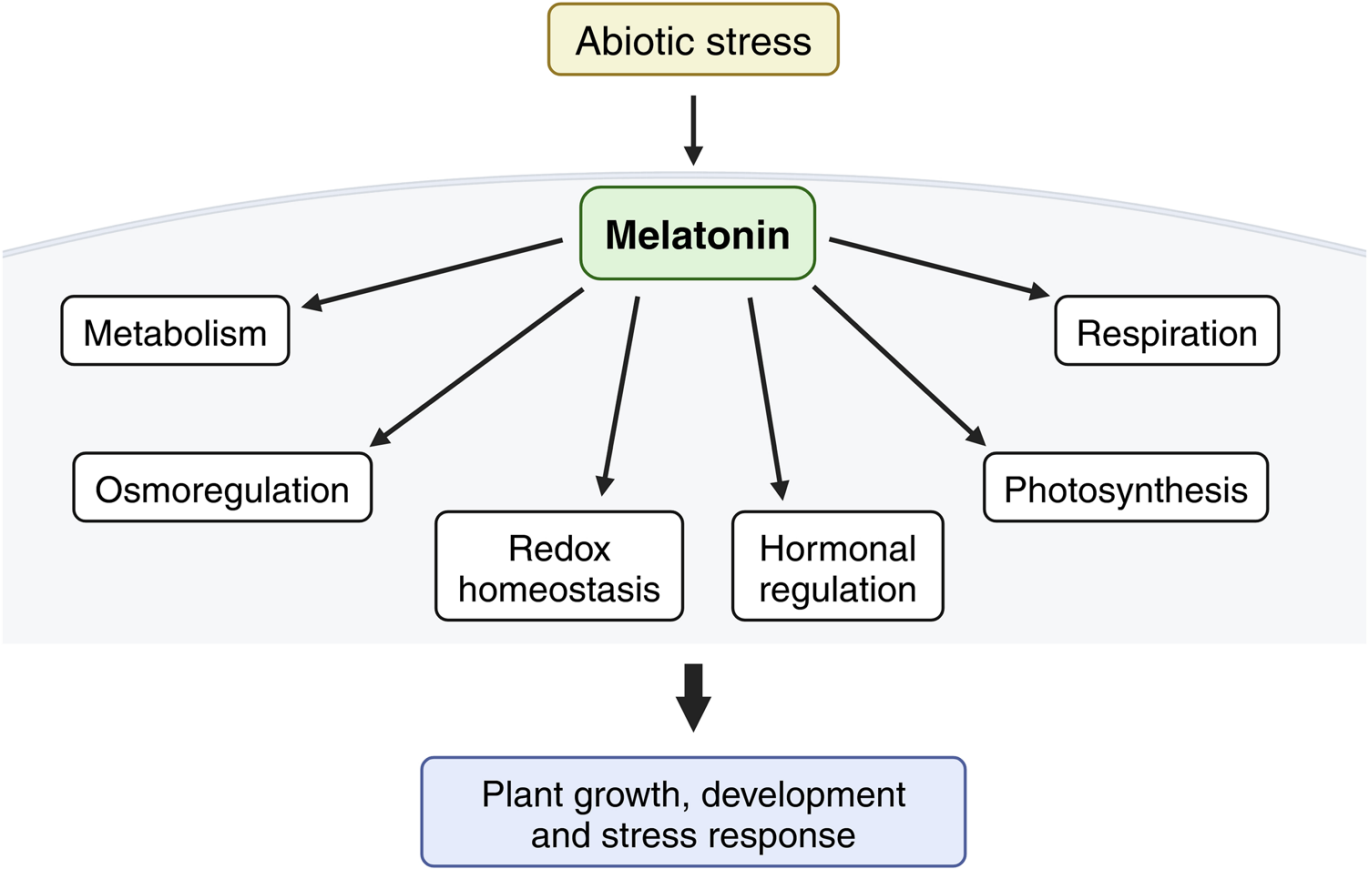
Model of melatonin action in plants in response to stress (figure drafted on the base of Arnao and Hernández-Ruiz 2021; Tiwari et al. 2021, using BioRender tool at http://biorender.com)

Melatonin signaling and its downstream effectors coordinate a variety of plant developmental processes, including seed germination, vegetative growth, flowering, and senescence (Tiwari et al. 2021). By influencing metabolites accumulation, osmoregulation, photosynthesis, mitochondrial respiration, redox homeostasis, and interactions with phytohormones, melatonin enables plants to fine-tune their responses to environmental stresses and ensure optimal growth (Fig. 3) (Sun et al. 2021; Gao et al. 2023).

Through its putative receptor, Cand2, mitogen-activated protein kinase (MAPK) cascades, and downstream transcription factors, melatonin influences the expression of genes related to plant growth, development, and stress responses (Gao et al. 2023). Notably, plant melatonin signaling system shares similarities with melatonin signal transduction in animals, where G-protein-coupled receptors (GPCRs) melatonin type 1 (MT1) and type 2 (MT2), activate a variety of signaling pathways, such as MAPK, protein kinase A, and calcium signaling. However, while animals possess two well-characterized GPCRs, studies indicate the existence of only one potential melatonin receptor in plants, which operates through a mechanism functionally similar to GPCRs (Hardeland 2009; Gao et al. 2022; Park 2024).

**Fig. 4:**
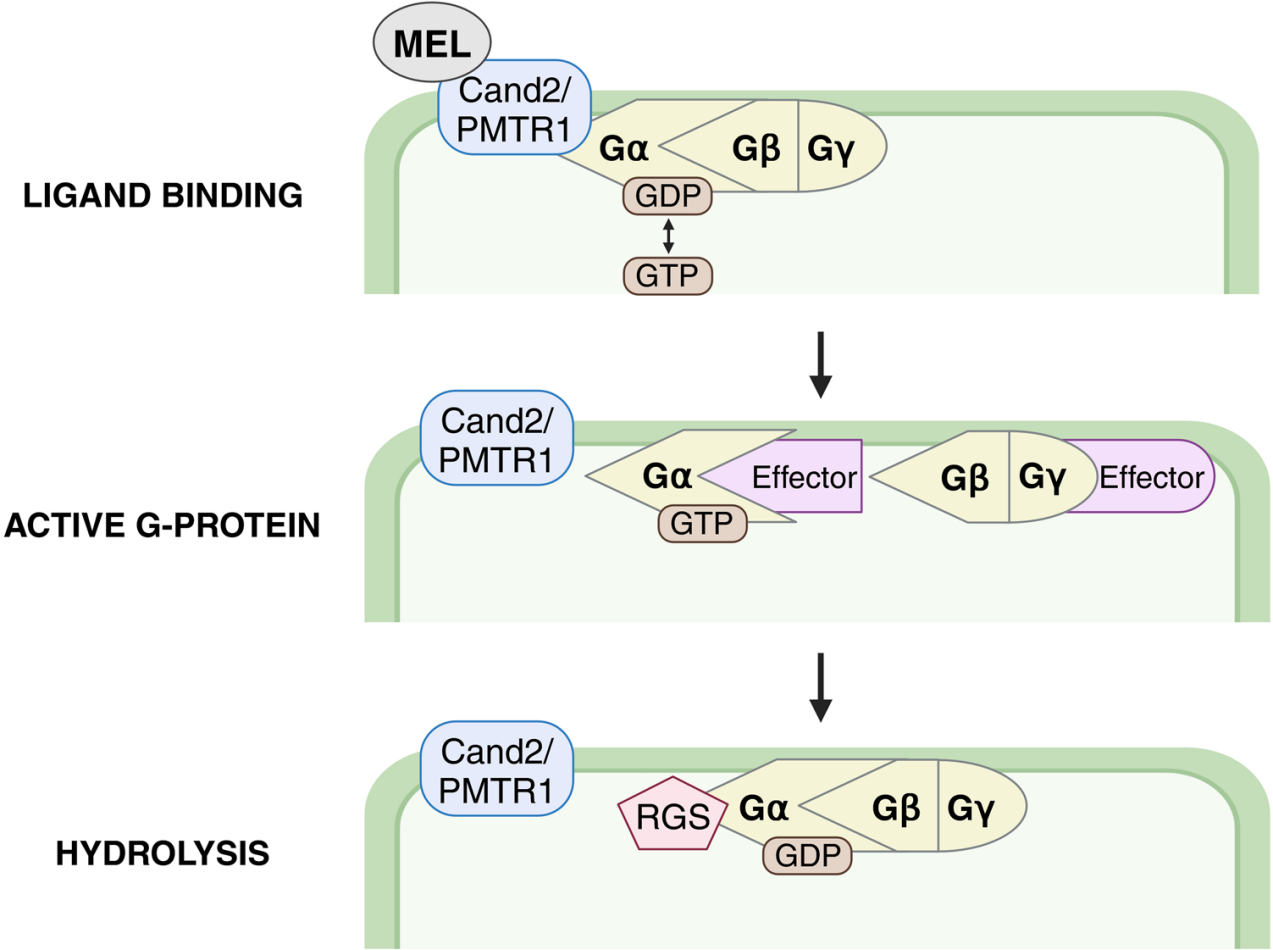
Proposed model for melatonin signaling via Cand2/PMTR1 in plants. Cand2/PMTR1, phytomelatonin receptor; Gα and Gβγ, G-protein subunits; GDP, guanosine diphosphate; GTP, guanosine triphosphate; MEL, melatonin; RGS, regulator of G-protein signaling protein (figure drafted on the base of Wei et al. 2018; Liu et al. 2021b, using BioRender tool at http://biorender.com)

Melatonin mediates its effects through the Cand2 protein, believed to be the first identified phytomelatonin receptor (PMTR1) in *A. thaliana* (Wei et al. 2018; Khan et al. 2023a). Cand2 is proposed to function as an uncanonical G-protein-coupled receptor (GPCR) due to its interaction with the G-protein α subunit (Gα). However, the activation mechanism remains unclear, largely because traditional G-protein signaling in plants differs from that in animals; in plants, Gα is self-activated (Jones et al. 2011). Liu et al. (2021b) suggested a model for non-canonical GPCR action in plants, incorporating aspects of G-protein signaling from both animals and plants (Fig. 4). In animals, GPCR interacts with a G-protein composed of Gα and a Gβγ dimer, with Gα bound to guanosine diphosphate (GDP). Upon ligand binding, the GPCR promotes the exchange of GDP for guanosine triphosphate (GTP), triggering the dissociation of Gα from the Gβγ dimer and initiating signaling. Gα, with its intrinsic GTPase activity, hydrolyzes GTP to GDP, completing the signaling cycle (Jiang et al. 2022). In turn, in plants, Gα spontaneously exchanges GDP, resulting in self-activation without the need for GPCR induction. The regulator of G-protein signaling protein hydrolyzes GTP, returning Gα to its inactive state. In the proposed model, Cand2 serves as an uncanonical GPCR, acting as a signaling molecule that induces Gα self-activation. Upon receiving a signal, Gα exchanges GDP for GTP and activates itself. The dissociated Gα and Gβγ dimer then interact with various intracellular effectors, such as transcription factors and enzymes, to regulate plant growth and responses to environmental stimuli (Liu et al. 2021b; Maruta et al. 2021).

In *A. thaliana*, Cand2 plays a critical role in melatonin-mediated stomatal closure, involving NADPH oxidase-dependent hydrogen peroxide production and the activation of Ca^2+^ channels (Wei et al. 2018). *A. thaliana* mutants lacking the *CAND2* gene are insensitive to melatonin-induced stomatal closure and exhibit increased sensitivity to osmotic stress, along with heightened ROS accumulation (Wang et al. 2021a). In wild-type plants, melatonin induces the expression of antioxidant enzymes such as catalase and superoxide dismutase in response to stress; however, this induction is suppressed in *CAND2* mutants. These findings underscore the essential role of Cand2 in melatonin-mediated gene expression and antioxidant responses. Bychkov et al. (2022) further demonstrated that melatonin, through Cand2, regulates mitochondrial gene expression during photooxidative stress. In *A. thaliana*, oxidative stress typically suppresses the expression of mitochondrial genes related to the respiratory chain, cytochrome c, ribosomal proteins, and transport proteins. Melatonin treatment during photooxidative stress contributed to maintain the expression of these genes in wild-type plants, but its effect on transcript accumulation in *CAND2* knockout mutants was minimal. These receptor-dependent effects highlight melatonin’s broad regulatory roles, leading to suggestions that melatonin could be classified as a new phytohormone (Arnao and Hernández-Ruiz 2019). Although knowledge about the putative melatonin receptor Cand2/PMTR1 primarily comes from studies on *A. thaliana*, an ortholog of the *CAND2* gene has been identified in several other plant species, including *O. sativa* (Barman et al. 2023), *Medicago sativa* (Yu et al. 2021), *Zea mays* (Wang et al. 2021b), *G. hirsutum* (Zhang et al. 2023b), and *Manihot esculenta* (Bai et al. 2022). However, no studies have yet been conducted on the role of Cand2/PMTR1 in cereal crops.

**Fig. 5:**
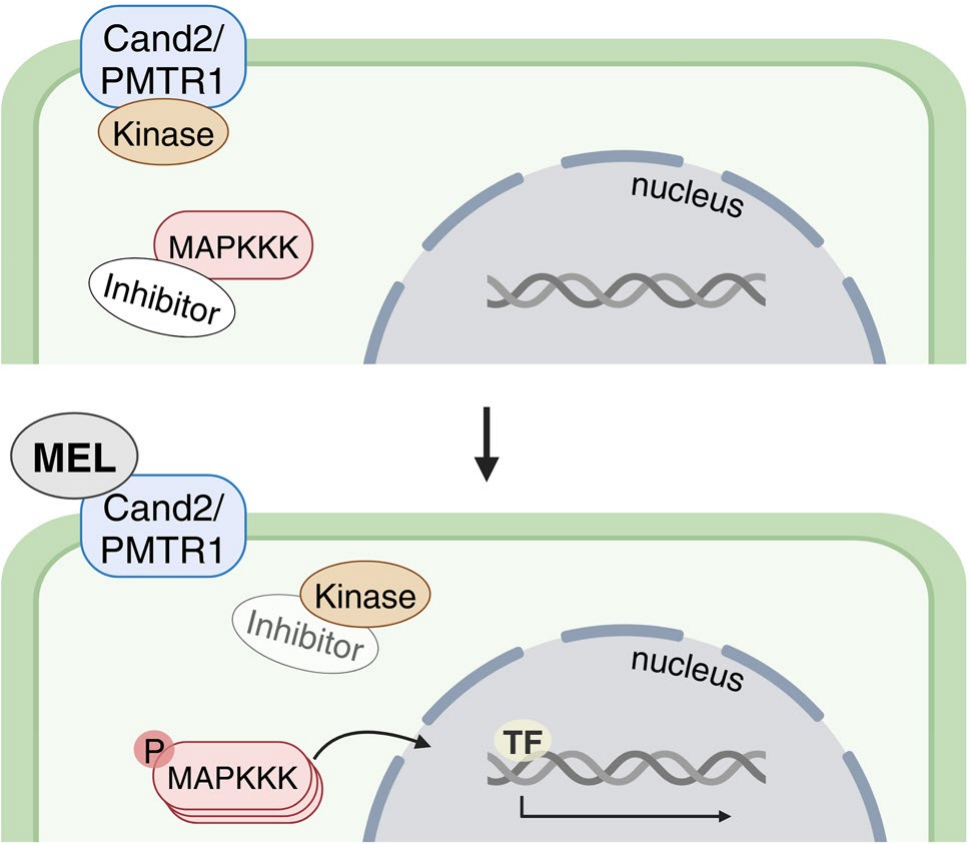
Proposed model of melatonin signal transduction through MAPK cascade. Cand2/PMTR1, phytomelatonin receptor; MAPKKK, mitogen-activated protein kinase; MEL, melatonin; TF, transcription factor (figure drafted on the base of Dzinyela et al. 2024; Liu et al. 2022a, b; Zhang and Zhang 2022, using BioRender tool at http://biorender.com)

A known intracellular effector activated by melatonin receptor binding in both plants and animals is MAPK, which converts signals into physiological responses. In the MAPK cascade, at least three kinases are sequentially phosphorylated: MAPKKK, MAPKK, and MAPK. Upon activation, the final kinase translocates within the cell to phosphorylate various targets, including transcription factors (Back 2021; Nikolaev et al. 2022; Zhang and Zhang 2022). While MAPKs function downstream of GPCR signaling in animals, studies have shown that melatonin-induced MAPK activation is impaired in *A. thaliana CAND2* mutants, but remains unaffected in both G-protein subunit α and β mutants. These findings suggest that melatonin signal transduction via MAPK may function independently of G-protein signaling (Chen et al. 2020; Lee and Back 2016; Yang et al. 2021b). Moreover, studies by Lee and Back (2016) have shown that melatonin-mediated MAPK activation in *A. thaliana* is not linked to calcium signaling. The independence of both calcium and G-protein signaling suggests that plants may have distinct melatonin signaling mechanisms compared to animals (Cecon et al. 2018). Liu et al. (2022b) proposed a model for melatonin signaling through the MAPK cascade, in which signaling remains inactive in the resting state due to a putative inhibitor that dephosphorylates MAPKKK (Fig. 5). Binding of melatonin to PMTR1 triggers the activation of the kinase responsible for inducing inhibitor ubiquitination. Following the degradation of the inhibitor, the MAPK cascade is activated, leading to the induction of various downstream effectors that allow melatonin to exert pleiotropic effects in plants. Further research on PMTR1 signaling via MAPK and the identification of new melatonin receptors will contribute to a better understanding of melatonin’s action in plants.

Recently, Slominski et al. (2023) identified two nuclear melatonin receptors in human cells: the aryl hydrocarbon receptor (AHR) and the peroxisome proliferator-activated receptor gamma (PPARγ). Binding to these receptors may explain the cytoprotective actions of melatonin, as both AHR and PPARγ have been extensively studied for their roles in promoting barrier function and inflammatory signaling (Konger et al. 2021; Rothhammer and Quintana 2019). To date, no nuclear melatonin receptors have been found in plants.

## Melatonin in antioxidant activity, redox homeostasis, and metabolic regulation

A primary function of melatonin, due to its chemical structure, is to act as a free radical scavenger. The antioxidant properties of melatonin derivatives prolong its effects in plants (Tan et al. 2014; Lee and Back 2022). The potential for an antioxidant cascade, where melatonin metabolites formed through interactions with free radicals continue to scavenge subsequent radicals, further enhances the antioxidative capacity of a single melatonin molecule (Reina and Martínez 2018). The stimulatory effect of melatonin on the activity of antioxidant enzymes, including superoxide dismutase, catalase, and peroxidase, has been documented in various crop species, such as *Prunus persica* (Gu et al. 2021), *Brassica napus* L. (Ali et al. 2023b), *T. aestivum* (Sun et al. 2023a), and *O. sativa* (Munir et al. 2024). Additionally, melatonin enhances the levels of ascorbic acid and glutathione, which are key antioxidants involved in the ascorbate–glutathione cycle, a major mechanism for hydrogen peroxide detoxification (Altaf et al. 2022a; Wang et al. 2012). Despite its antioxidant effects, melatonin stimulates respiratory burst oxidase homologs (RBOH), enzymes that transfer electrons from NADPH to oxygen, leading to the production of superoxide anions (Gu et al. 2022). While the excessive accumulation of ROS can cause oxidative damage and plant cell death, these molecules also function as signaling agents, enabling plants to adapt to dynamic environments (Khan et al. 2023b). The regulatory mechanism of melatonin-mediated ROS signaling has been experimentally validated in *Solanum lycopersicum* (Gong et al. 2017), *A. thaliana* (Li et al. 2020), and *N. tabacum* (Xiao et al. 2023). By acting as both a free radical scavenger and a mediator in antioxidant and pro-oxidant pathways, melatonin significantly contributes to maintaining redox homeostasis in plants. The interplay between ROS, reactive nitrogen species, and melatonin, including the induction of melatonin biosynthesis during stress conditions, forms a complex redox network essential for plant functioning (Arnao and Hernández-Ruiz 2019).

The action of melatonin in plants is linked with various components of the redox network, including nitric oxide (NO) (Zhu et al. 2019); however, studies in this area remain limited. Curiously, researches using human cell lines suggest that the interaction between melatonin and NO plays a role in regulating mitochondrial homeostasis. For instance, Sarti et al. (2013) proposed a model in which melatonin, at low concentrations, stimulates the expression of nitric oxide synthase (NOS), leading to an increase in NO levels and a reduction in oxidative phosphorylation efficiency. At higher concentrations, melatonin interacts with calmodulin, inhibiting NOS activity and reducing NO production in mitochondria. Moreover, melatonin inhibits mitochondrial permeability transition pores and induces uncoupling proteins, which helps maintain the optimal membrane potential and enhances oxidative phosphorylation (Slominski et al. 2017b; Tan et al. 2016b). Interestingly, studies in human cell lines indicate also that melatonin promotes DNA repair processes. Santoro et al. (2013) revealed that melatonin activates a p53-mediated DNA damage response, thereby influencing genome integrity. Subsequently, it was shown that melatonin induces activity of nucleotide excision repair factors, XPC and XPA, in a manner independent of MT1 and MT2 receptors (Janjetovic et al. 2017). Unfortunately, both the potential mechanism of melatonin-mediated DNA repair in plants and the role of the interactions between melatonin, NO, and calmodulin in mitochondrial regulation have yet to be clarified.

Melatonin regulates various metabolic processes in plants, including the accumulation of key metabolites in response to stress signals (Arnao and Hernández-Ruiz 2019). During drought stress, melatonin treatment enhances the levels of osmoprotectants such as polyamines, proline, and soluble sugars in various crop species (Tiwari et al. 2021). These osmoprotectants help stabilize cell membranes, maintain proper cell turgor, and prevent organelle damage, while also influencing stomatal arrangement, thereby affecting plant photosynthetic capacity (Ozturk et al. 2021). Through its regulation of polyamine metabolism, melatonin further boosts the antioxidant system, as polyamines possess direct antioxidant properties (Tiwari et al. 2023). Additionally, melatonin promotes the accumulation of flavonoids, anthocyanins, and carotenoids, which contribute to plant protection against abiotic stresses by absorbing UV light and scavenging ROS (Zhang et al. 2016; Liang et al. 2018; Sharma et al. 2020). Long-term UV radiation exposure can lead to cellular damage and elevated ROS levels, resulting in oxidative stress in plants. Melatonin-induced biosynthesis of flavonoids and anthocyanins serves as a protective mechanism, shielding cellular organelles from the effects of intense light (Ferreyra et al. 2021; Pan et al. 2023). Sun et al. (2023b) demonstrated that anthocyanin accumulation plays a crucial role in melatonin-mediated chromium stress tolerance in *S. lycopersicum.* Acting as metal chelators, anthocyanins reduce chromium toxicity by sequestering it into vacuoles. These findings suggest that by increasing the concentration of osmotic solutes and high-value metabolites, melatonin enables plants to effectively counteract stress signals.

## Melatonin in regulation of photosynthesis and mitochondrial respiration

Numerous studies have shown that melatonin positively influences photosynthesis under adverse environmental conditions (Szafrańska et al. 2017; Yang et al. 2021c). The photosynthetic system is highly sensitive to abiotic stresses, which can damage thylakoid membranes, reduce photosynthetic rates, and inhibit plant growth (Muhammad et al. 2021). The concurrent occurrence of photosynthesis and melatonin production in chloroplasts is considered a complex defense mechanism that maintains photosynthetic performance during stress (Pan et al. 2023).

Jahan et al. (2020) reported that melatonin treatment in response to nickel-induced phytotoxicity increases the expression of chlorophyll synthesis genes, enhances gas exchange parameters, and improves the photochemical activity of Photosystem II (PSII) in *S. lycopersicum*. Similarly, Sharma et al. (2020) found that melatonin enhances photosynthesis in *Carya cathayensis* during drought stress by inducing metabolic pathways associated with chlorophyll synthesis and downregulating genes responsible for chlorophyll degradation. In *C. sativus*, melatonin upregulates proteins related to PSII and PSI, increases the activity of their reaction centers, and improves photosynthetic electron transfer (PET) efficiency in response to chilling stress. It has also been shown that melatonin increases the abundance of proteins involved in carbon fixation (Zhang et al. 2021). Altaf et al. (2022b) demonstrated that melatonin enhances photosynthesis under cold stress in *C. annuum*, improving gas exchange, increasing photosynthetic pigment content, and upregulating genes related to PSI and PSII. Moreover, melatonin increases the activity of photosynthetic enzymes in the Calvin-Benson cycle, enabling plants to maintain high carbon assimilation capacity. These findings clearly demonstrate melatonin’s critical role in maintaining photosynthetic efficiency during stress by promoting pigment recovery, improving gas exchange parameters, and regulating genes related to PSI, PSII, and PET.

In contrast to the extensive research on photosynthesis regulation, fewer studies have examined the effects of melatonin on mitochondrial respiration in plants. Samanta et al. (2020) reported that, under drought conditions in *O. sativa*, melatonin increases the activity and gene expression of key respiratory enzymes such as pyruvate dehydrogenase (PDH) and citrate synthase, indicating its role in regulating the Krebs cycle rate. Similarly, Turk and Genisel (2020) observed that melatonin has a comparable effect in *Z. mays* under cold stress, demonstrating that melatonin enhances the gene expression and protein levels of enzymes related to the mitochondrial electron transport chain (ETC). By increasing the activity of cytochrome oxidase, alternative oxidase, and adenosine triphosphate (ATP) synthase, melatonin increases ATP production, which is essential for maintaining cellular processes. These findings align with those of Bychkov et al. (2022), who revealed that during photooxidative stress in *A. thaliana*, melatonin induces the expression of genes related to various respiratory chain complexes and cytochrome c biosynthesis. Overall, melatonin influences mitochondrial respiration in plants in various environmental conditions by regulating the gene expression of PDH, enzymes associated with the Krebs cycle, and the mitochondrial ETC.

## Melatonin in phytohormonal regulation

Early research on the interaction between melatonin and phytohormones focused on auxins due to the structural similarly between melatonin and indole-3-acetic acid (IAA), as well as melatonin’s auxin-like effects in promoting plant vegetative growth. Studies revealed that melatonin promotes auxin responses by regulating a wide range of genes involved in auxin biosynthesis and its signaling pathways (Khan et al. 2022). Wei et al. (2022) reported that melatonin enhances the expression of genes related to auxin biosynthesis (*YUCCA*), signal transduction (*TIR1*, *AFB3)*, transport (*AUX1*, *PIN*), and various auxin-responsive genes in *G. max* (Table 2). Similarly, Liang et al. (2017) demonstrated that melatonin-induced root growth in *O. sativa* is associated with the activation of genes involved in the auxin signaling pathway. Therefore, melatonin regulates plant growth at least partially through an auxin-dependent manner.

**Table 2:** The effect of melatonin on phytohormones in crops

Hormone	Crop species	Melatonin interaction	Gene expression^a^	References
Auxin	*Oryza sativa*	Synergistic	**↑***Aux/IAA*, *ARF*, *SAUR*, *GH3*	Liang et al. (2017)
	*Malus prunifolia*	Synergistic	**↑***YUCCA1*, *YUCCA10*, *ARF7*,* ARF19, AUX1, PIN1, PIN3, GH3*	Mao et al. (2020)
	*Glycine max*	Synergistic	**↑***YUCCA*, *AMI1, TIR1/AFB, Aux/IAA, ARFs*, *AUX1/LAX, PIN/PIL, SAUR-like, GH3*	Wei et al. (2022)
Cytokinin	*Lolium perenne*	Synergistic	**↑***IPT2*,* LOG1, ARR1, ARR10*	Zhang et al. (2017)
	*Agrostis stolonifera*	Synergistic	**↑***IPT, BRR2, ARR4, ARR9, BRR10, HK3, HK4, HP1, HP4*	Ma et al. (2018)
Abscisic acid	*Lolium perenne*	Antagonistic	↓ *ZEP*, *NCED1*, *ABI3*, *ABI5*	Zhang et al. (2017)
	*Solanum lycopersicum*	Antagonistic	↓ *NCED1*, *NCED2*, *AAO3*, *ABI3*, *ABI5* **↑***CYP707A1*, *CYP707A2*	Jahan et al. (2021)
	*Oryza sativa*	Antagonistic	↓ *NCED1*, *NCED2*, *AAO, ABI5*	Li et al. (2021b)
	*Cucumis sativus*	Antagonistic	↓ *NCED2* **↑***CYP707A1, PYR/PYL/RCAR,, SnRK2, PP2C3, PP2C5*	Zhang et al. (2023a)
Gibberellin	*Cucumis sativus*	Synergistic	**↑***GA20ox, GA3ox*	Zhang et al. (2014)
	*Solanum lycopersicum*	Synergistic	**↑***GA20ox1*, *GA20ox2* ↓ *GAI, GA2ox1*, *GA2ox2*	Jahan et al. (2021)
	*Oryza sativa*	Synergistic	**↑***GA1* ↓ *GA2ox1*, *GA2ox2*, *GA2ox3*, *GA2ox4*	Li et al. (2021b)
Jasmonic acid	*Solanum lycopersicum*	Synergistic	**↑***AOC, LoxD, PI II* ↓ *JAZ1*	Liu et al. (2019)
	*Brassica napus*	Antagonistic	**↑***JAZ* ↓ *AOC*	Tan et al. (2019)
	*Triticum aestivum*	Synergistic	**↑***LOX1.5*, *LOX2*	Luo et al. (2023)
Salicylic acid	*Solanum lycopersicum*	Synergistic	**↑***PR1, PR5*	Zhao et al. (2019a, b)
	*Actinidia deliciosa*	Synergistic	**↑***PAL*, *BA2H*, *PR1*	Guo et al. (2023)
Ethylene	*Musa acuminata*	Antagonistic	↓ *ACO1, ACS1*	Hu et al. (2017)
	*Pyrus communis*	Antagonistic	↓ *ACO1, ACS1*	Zhai et al. (2018)
	*Solanum lycopersicum*	Synergistic	**↑***ACS2*, *ACS4*, *ACO1*, *ACO3*, *ETR1*, *ETR3*, *ETR4*, *ETR6*	Sun et al. (2020a, b)
	*Vitis vinifera*	Antagonistic	↓ *ACO2*, *ERF4*, *ERF5*, *ERF17*, *ERF26*, *ERF53*,* ERF71*	Liu et al. (2021a)
	*Malus domestica*	Synergistic	**↑***ACS1*, ACS3, *ACO1*,* ACO2*	Verde et al. (2023)
	*Triticum aestivum*	Antagonistic	↓ *ACS*	Sehar et al. (2023)
Brassinosteroid	*Oryza sativa*	Synergistic	**↑***DWF4*, *BRI1*, *RAVL1*, *D11, XTR3* ↓ *BZR1*	Hwang and Back (2018)
	*Brassica napus*	Synergistic	**↑***SQE, STE1* ↓ *BSK*	Tan et al. (2019)

^a^The upward arrow represents an increase in gene expression caused by melatonin, whereas the downward arrow indicates a decrease

The effect of melatonin in plants has also been associated with transcriptional changes in genes related to cytokinins (CKs). Ma et al. (2018) demonstrated that melatonin increases the expression of CK biosynthesis genes (*IPT*) and elements of its signaling pathway in *Agrostis stolonifera*, delaying drought-induced leaf senescence and improving various physiological parameters. Similarly, Zhang et al. (2017) observed that melatonin, by modulating levels of CKs and abscisic acid (ABA), suppresses heat-induced leaf senescence in *Lolium perenne* L. During heat stress, melatonin induces the expression of genes involved in cytokinin biosynthesis (*IPT2*, *OG1*) and signaling pathways, while reducing the expression of genes associated with ABA biosynthesis (*ZEP*, *NCED*) and its transcription factors (*ABI3*, *ABI5*) (Table 2). The role of melatonin in mediating stress responses via ABA-dependent pathways has been extensively studied in plants (Ali et al. 2023a). Jahan et al. (2021) demonstrated that melatonin treatment affects ABA and gibberellin (GA) levels in *S. lycopersicum* during heat stress. In response to stress signals, melatonin decreased the expression of genes related to ABA biosynthesis and signaling while inducing ABA catabolism (Table 2). Melatonin-treated plants also exhibited increased expression of GA biosynthetic genes** (***GA20ox1*, *GA20ox2*) and reduced expression of genes involved in GA catabolism (*GA2ox1*, *GA2ox2*) and signaling inhibition (*GAI*)*.* Similar findings were reported by Li et al. (2021b), who showed that melatonin, in response to low-temperature stress (LT), induces GA biosynthesis and reduces ABA accumulation during *O. sativa* seed germination. Additionally, Zhang et al. (2023a) examined the effects of melatonin on *C. sativus* seed germination under LT stress, highlighting its influence on the expression of genes involved in ABA signaling. Zhang et al. (2014) also demonstrated that melatonin induces GA biosynthesis under salt stress in *C. sativus* seeds, promoting germination. In *C. cathayensis*, Sharma et al. (2020) revealed that melatonin reduces ABA accumulation while upregulating the levels of phytohormones such as GA, CK, and BR, contributing to enhanced drought tolerance.

Studies indicate that melatonin regulates both jasmonic acid (JA) and salicylic acid (SA) levels in plants. The regulation of JA and SA biosynthesis is critical for plant development, as many downstream effectors of their signaling pathways are involved in stress responses (Wang et al. 2020; Prakash et al. 2021). Guo et al. (2023) reported that melatonin induces the expression of enzymes involved in SA biosynthesis (*PAL*, *BA2H*) in *Actinidia deliciosa*, leading to the upregulation of SA-responsive genes (*PR1*) and enhancing plant resistance to chilling and oxidative stress. These findings align with Esmaeili et al. (2023), who observed increased SA levels and PAL activity in *Linum album* following melatonin treatment. Similarly, Zhang et al. (2019) demonstrated that melatonin induces the accumulation of SA and the expression of SA-responsive genes (*PR1*, *PR5*) in *S. lycopersicum*. Similarly, Luo et al. (2023) demonstrated that melatonin enhances the expression of JA biosynthetic genes (*LOX1.5*, *LOX2*) in *T. aestivum* during drought stress (Table 2). Additionally, melatonin treatment increased methyl jasmonate (MeJA) levels in *C. lanatus* in response to cold stress (Li et al. 2021a). Liu et al. (2019) demonstrated that melatonin stimulates MeJA accumulation in *S. lycopersicum* plants by upregulating key JA biosynthetic enzymes (LoxD, AOC) and repressing the negative regulator of JA signaling (JAZ). Conversely, in *B. napus* seedlings under high salinity conditions, melatonin reduced JA accumulation, alleviating stress-induced growth inhibition (Tan et al. 2019).

Ethylene (ET), like JA and SA, modulates plant defense mechanisms, enabling precise responses to environmental stressors. Additionally, ET plays a regulatory role in various physiological processes, including plant senescence (Chen et al. 2021). Studies on the ripening of *Musa acuminata* L. (Hu et al. 2017) and *Pyrus communis* L. fruits (Zhai et al. 2018) demonstrated that melatonin treatment represses ethylene biosynthesis by downregulating *ACS1* and *ACO1*. In contrast, Verde et al. (2023) revealed that melatonin increases ET content during the ripening of *Malus domestica* fruits by enhancing the expression of its biosynthetic genes (Table 2). Moreover, Sun et al. (2020b) demonstrated that melatonin induces ET signaling in *S. lycopersicum* fruits, leading to increased carotenoid levels. Under abiotic stress, Liu et al. (2021a) found that melatonin mitigates the adverse effects of ozone stress in *Vitis vinifera* L. by inhibiting ET biosynthesis and signaling. Similarly, melatonin downregulates ET synthesis in *T. aestivum*, improving plant resistance to high temperatures (Sehar et al. 2023). These findings suggest that melatonin regulates ET accumulation and signaling, adjusting its levels based on physiological processes and environmental conditions.

Studies on the interaction between melatonin and strigolactones (SLs) or BRs in plants are limited. Zhang et al. (2019) suggested that SLs inhibit melatonin biosynthesis during floral transitions in *A. thaliana*, promoting flowering, though the exact mechanism remains unclear. Regarding BRs, Lee and Back (2019) demonstrated that suppressing melatonin biosynthesis genes (*SNAT2*, *T5H*, and *COMT*) in *O. sativa* led to a dwarf phenotype characterized by erect leaves and reduced expression of *DWARF4*, a key gene involved in BR biosynthesis. Moreover, Hwang and Back (2018) revealed that melatonin and BR interaction in *O. sativa* seedlings is associated with skotomorphogenesis. These findings suggest that melatonin coordinates plant development by regulating BR biosynthetic genes. Additionally, melatonin promotes the expression of genes involved in BR biosynthesis and signaling (Table 2). Tan et al. (2019) observed that melatonin treatment in *B. napus* upregulates genes involved in campesterol synthesis (*SQE*, *STE1*), a precursor of brassinolide, while downregulating one of the two BR receptor substrates (*BSK*). Moreover, Hwang and Back (2022) demonstrated that reducing melatonin levels in *O. sativa*, due to the suppression of *RAVL1* and *Gα* genes, was partially alleviated by exogenous BR treatment, indicating mutual regulation between melatonin and BR synthesis, possibly mediated by a common signaling ligand involving Gα.

## Melatonin-phytohormone crosstalk in root development

Abiotic stresses, such as drought, significantly affect root growth and structure, reducing the root’s ability to absorb water and nutrients from the soil. To cope with these environmental challenges, plants adapt by modifying their root system architecture, often directing root growth toward areas with greater water availability while avoiding dry surface soils where drought stress is most severe (Karlova et al. 2021). This adaptive response involves the activation of various signaling pathways in roots, many of which are associated with phytohormones. Understanding the molecular mechanisms behind these root adaptations is crucial for developing strategies to enhance crop resilience and productivity under stress conditions (Nziengui and Ditengou 2023). The role of melatonin in root development was first suggested in the early 2000s by Murch et al. (2001), who observed a correlation between endogenous melatonin levels and de novo root formation in *Hypericum perforatum* L. Since then, substantial evidence has demonstrated that melatonin regulates both the number and length of adventitious and lateral roots in various plant species (Arnao and Hernández-Ruiz 2017, 2021). Despite this emerging role, the precise molecular mechanisms and downstream targets of melatonin in root development remain unclear (Sun et al. 2021).

Melatonin-mediated root growth is often associated with its interaction with auxins. Liang et al. (2017) revealed that melatonin treatment promotes the number and length of lateral roots in *O. sativa* by activating auxin signaling. Ren et al. (2019) proposed a model illustrating the interaction between melatonin and auxin in lateral root formation in *A. thaliana*. According to this model, melatonin regulates the levels of the PIN5 protein, which mediates auxin transport from the endoplasmic reticulum to the nucleus. Both auxin and melatonin treatments downregulate *PIN5* expression in roots, possibly through the WAG1 kinase, which reduces auxin transport into the nucleus and increases cytosolic auxin levels, thereby promoting lateral root growth. In another study, Yang et al. (2021a) demonstrated that melatonin stimulates primary root growth in *A. thaliana* through an IAA-dependent mechanism by modulating the expression of genes involved in auxin biosynthesis. Melatonin and IAA regulate a similar set of genes in *A. thaliana* roots, and the effect of melatonin on primary root growth diminished when auxin biosynthesis inhibitors were present. Moreover, in *Malus prunifolia*, melatonin influences adventitious root (AR) growth by increasing IAA levels and inducing the expression of *WUSCHEL-related Homeobox 11* (*WOX11*) in the AR formation zone. The expression of *WOX11* is also regulated by the auxin signaling pathway, suggesting that WOX11 may serve as a key mediator in the melatonin-auxin interaction during root primordia development (Mao et al. 2020; Zhang et al. 2018).

**Fig. 6:**
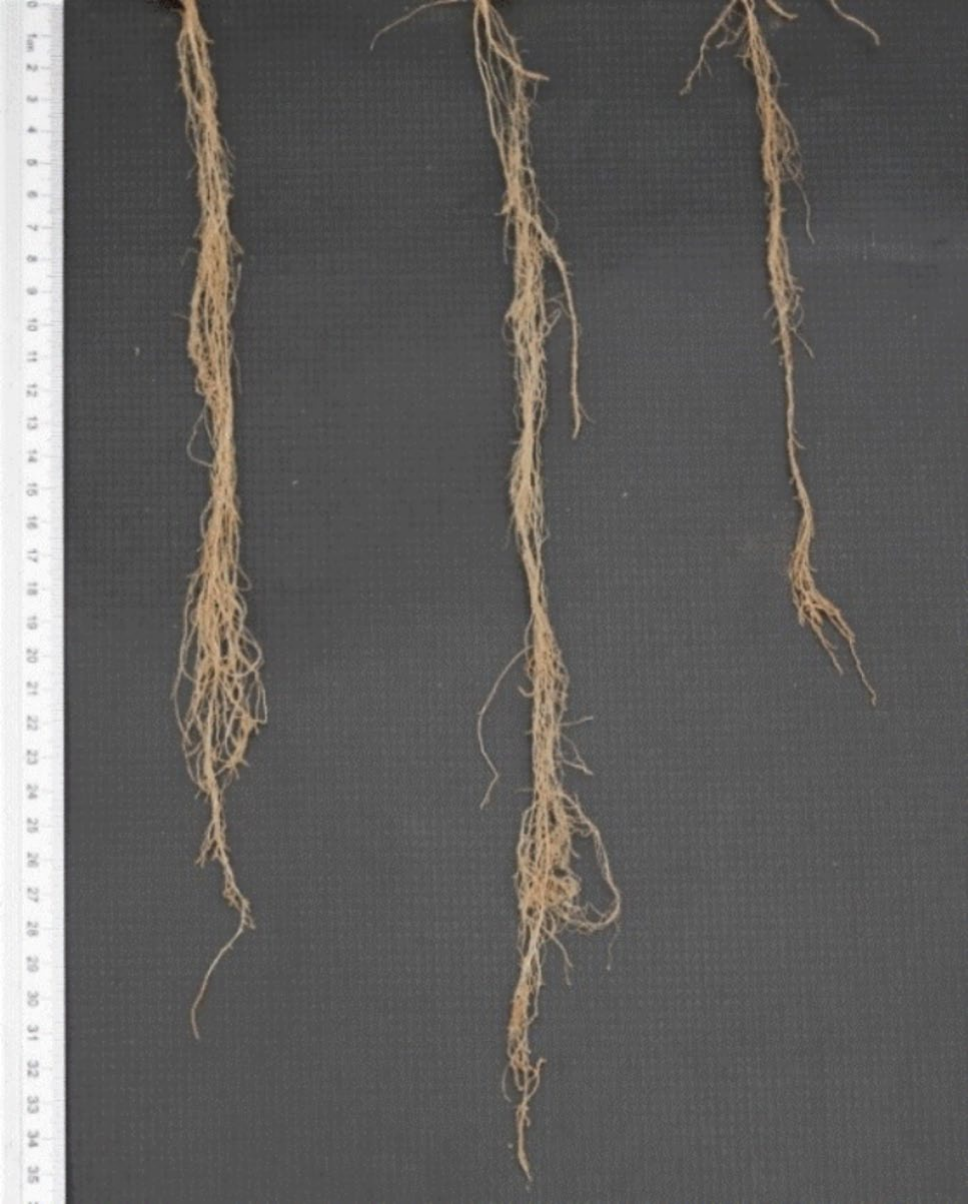
Differences in barley root length: BR-deficient mutant BW091, brh3.g (left), wild-type barley cv. Bowman (middle) and BR-insensitive plant BW885, uzu1.a (right) (photo by M. Michałek)

Melatonin regulates the levels of various phytohormones beyond auxins in plant root systems. Duan et al. (2022) revealed that melatonin increases the levels of IAA, BR, and GA while reducing ABA content in *G. hirsutum* seedling roots, alleviating the growth inhibition caused by salt stress. Similarly, Ma et al. (2023) reported that melatonin enhances hormone-mediated root growth in *H. vulgare* roots under low phosphorus stress, showing that melatonin treatment increases IAA and GA levels while decreasing ABA, thereby influencing root length. Similarly, melatonin elevates IAA and GA levels and lowers ABA concentration in *M. prunifolia* roots, promoting AR formation (Mao et al. 2020). Additionally, Hu et al. (2020) demonstrated that melatonin stimulates *Cucumis melo* L. root growth under copper stress by reducing JA biosynthesis. In addition to auxins, BRs are key regulators of lateral and AR formation. BRs influence root length by controlling cell elongation and meristem size (Jaillais and Vert 2016). Plants with BR deficiencies or impaired BR signaling exhibit significantly shortened root phenotypes (Fig. 6). While BRs interact with auxins and other compounds during root growth, many of these interactions remain poorly understood (Altamura et al. 2023). Further investigation is required to unravel the complexities of BR involvement in root development and their interplay with other phytohormones.

Recent studies highlight that both melatonin and BRs regulate plant root growth by influencing the expression of cell cycle genes. Moreover, melatonin influences lateral root growth in *S. lycopersicum* by stimulating enzymes such as polyamine oxidase (PAO) and RBOH, leading to the production of ROS, including hydrogen peroxide and superoxide radicals. These ROS influence the expression of cell cycle genes such as cyclin-dependent kinase A1 (*CDKA1*) and cyclin D3 (*CYCD3*) (Chen et al. 2019). The study correlated melatonin-induced lateral root formation to the upregulation of *PAO* and *RBOH* genes in the lateral root emerging zone, suggesting these genes act downstream of melatonin. Tian et al. (2024) further validated these findings in tomato roots, demonstrating that melatonin modulates root architecture by interacting with endogenous BRs. Similarly, Wen et al. (2023) showed that 24-epibrassinolide (EBL) induces AR formation in *C. annuum* via the PAO- and RBOH-dependent production of ROS. Through gene family screening and expression analysis, a correlation was observed between EBL-induced AR formation and the upregulation of various PAO and *RBOH* genes in the roots. Silencing these genes reduced ROS accumulation and suppressed EBL-induced root formation. Furthermore, EBL treatment influenced the expression of cell cycle-related genes, such as *CYCD3* and *CDKA1*, in a ROS-dependent manner. These findings suggest that ROS signals generated by both EBL and melatonin activate signaling pathways that upregulate the expression of cell cycle genes, prompting root formation and elongation.

**Fig. 7:**
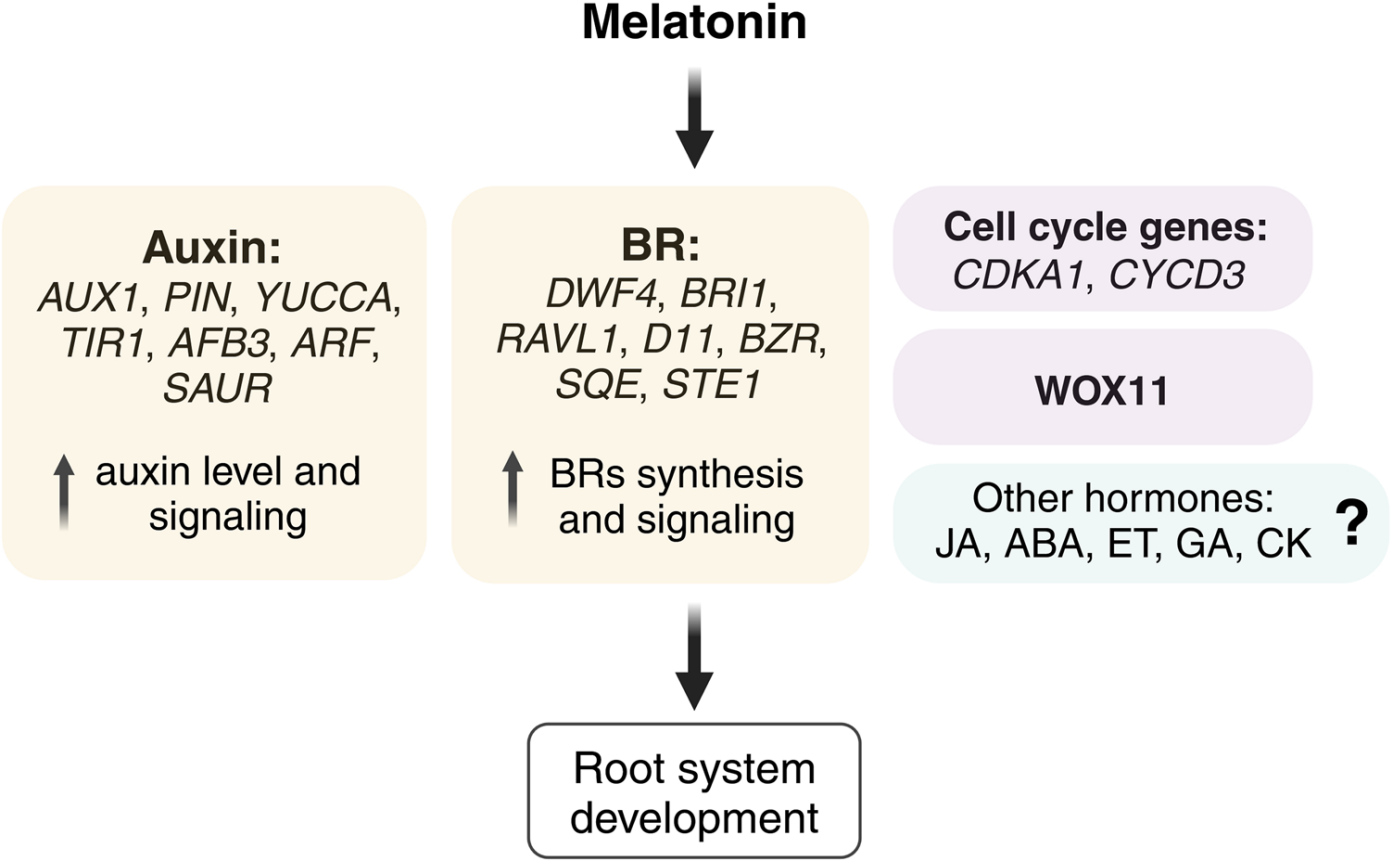
Regulation of root growth by melatonin through various target pathways (figure drafted on the base of Chen et al. 2019, Hwang and Back 2018, Mao et al. 2020, Tan et al. 2019, Wei et al. 2022, using BioRender tool at http://biorender.com)

Auxins and brassinosteroids likely act as key co-regulators of root growth alongside melatonin (Fig. 7). However, the interaction between melatonin and other hormones, such as GA, CK, and ABA, in the root system remains unexplored. Further research is needed to elucidate the molecular mechanisms underlying melatonin crosstalk with phytohormones and its role in regulating cell cycle genes and *WOX11* expression during root development.

## Conclusions and future directions

As evidenced, melatonin holds significant potential as a master regulator of various aspects of plant functioning, including its role in mitigating the harmful effects of stress. Its interactions with phytohormones hold significant promise for enhancing plant resilience to environmental stressors, as these phytohormones regulate various physiological and developmental processes. A viable strategy to support sustainable crop production under drought conditions involves leveraging melatonin’s interactions with phytohormones such as auxins and BRs to stimulate root growth and enhance water and nutrient absorption from the soil. The identification of ligands involved in melatonin-auxin crosstalk, such as *PIN5* and *WOX11*, in root development offers new opportunities to optimize crop root systems. Additionally, genetic studies have identified potential ligands in melatonin-BR interactions, including *SNAT2*, *DWF4*, *Gα*, *CDKA*, and *CYCD3* genes. However, the regulatory role of melatonin in shaping root architecture remains controversial, and the molecular mechanisms of its crosstalk with phytohormones are still not fully understood. The recent discovery of the receptor PMTR1/Cand2 could enhance understanding of melatonin’s involvement in hormonal signaling pathways. Additional research on crop plants using exogenous melatonin, as well as melatonin-deficient and -insensitive mutants, is essential to unravel the complex regulatory network of melatonin in plants.

Despite the surge in melatonin research, numerous aspects of its catabolism, signaling, phytohormone crosstalk, and its role in plant stress tolerance remain unclear. Further research should prioritize several key areas to advance understanding of melatonin’s role in plants. First, the role of Cand2/PMTR1 in other crop species, particularly in cereals, warrants investigation and investigating its involvement in the activation of G-protein signaling and the MAPK cascade could provide valuable insight. Another important area is the function of major melatonin metabolites in crop plants, as this remains poorly understood. Research should also aim to determine whether enzymatic or non-enzymatic melatonin transformation is more prevalent in plants. Another promising avenue is the interaction between melatonin and nanoparticles, as well as non-coding RNAs, including microRNAs. Finally, identifying new target genes for melatonin and potential ligands involved in its interaction with phytohormones will be critical for gaining a deeper insight into the regulatory functions of melatonin in plant biology. Given that research on melatonin in cereals is still in its infancy, significant efforts are needed, with barley emerging as a promising model species for advancing our understanding in this crop group.

## Abbreviations

AAO: Abscisic aldehyde oxidase
ABI5: Abscisic acid-insensitive 5
ACO: 1-Aminocyclopropane-1-carboxylate oxidase
ACS: 1-Aminocyclopropane-1-carboxylate synthase
AMI1: Amidase 1
AOC: Allene oxide cyclase
ARFs: Auxin response factors
ARR: A-type response regulator
Aux/IAA: Auxin/indole-3-acetic acid related genes
AUX1: Auxin resistant 1
BA2H: Benzoic acid-2-hydroxylase
BRI1: Brassinosteroid insensitive 1
BRR: B-type response regulator
BSK: Brassinosteroid-signaling kinase
BZR1: Brassinazole resistant 1
CYP707A1: Abscisic acid 8′-hydroxylase 1
CYP707A2: Abscisic acid 8′-hydroxylase 2
D11: Dwarf 11
DWF4: Dwarf 4
ERF: Ethylene-responsive transcription factor
ETR: Ethylene receptor
GA20ox: Gibberellin 20-oxidase
GA2ox: Gibberellin 2-oxidase
GA3ox: Gibberellin 3-oxidase
GA1: Gibberellin A1
GAI: Gibberellic-acid insensitive
GH3: Gretchen hagen 3
HK: Histidine kinase
HP: Histidine phosphotransfer
IPT: Isopentenyl transferase
JAZ: Jasmonate zim domain 1
LOX: Lipoxygenase
LoxD: Lipoxygenase D
NCED1: 9-Cis-epoxycarotenoid dioxygenase 1
LOG1: Lonely guy 1
PAL: Phenylalanine ammonia lyase
PI II: Proteinase inhibitor II
PILs: Pin-like genes
PINs: Pin-formed genes
PP2C: Protein phosphatase type 2C
PR1: Pathogenesis-related gene 1
PYR/PYL/RCAR: Pyrabactin resistance/pyrabactin resistance 1-like/regulatory component of ABA receptor
RAVL1: Related to abi3/vp1-like 1
SAUR: Small auxin up-regulated RNA genes
SnRK: Snf1-related kinase
SQE: Squalene epoxidase
STE1: ∆7-Sterol-c5-desaturase
TIR1/AFB: Transport inhibitor response 1/auxin signaling f-box protein
XTR3: Xyloglucan endotransglycosylase related 3
YUCCA: Flavin-dependent monooxygenase
ZEP: Zeaxanthin epoxidase
